# Comparative Analysis of NanoLuc Luciferase and Alkaline Phosphatase Luminescence Reporter Systems for Phage-Based Detection of Bacteria

**DOI:** 10.3390/bioengineering9090479

**Published:** 2022-09-16

**Authors:** Shalini Wijeratne, Arindam Bakshi, Joey Talbert

**Affiliations:** Department of Food Science and Human Nutrition, Iowa State University, Ames, IA 50011, USA

**Keywords:** alkaline phosphatase, NanoLuc, luciferase, luminescence, phage, reporter, bacteriophage, *E. coli*

## Abstract

Reporter phage assays are a promising alternative to culture-based assays for rapidly detecting viable bacteria. The reporter systems used in phage-based detection are typically enzymes and their corresponding substrates that provide a signal following infection and expression. While several reporter systems have been developed, comparing reporter systems based on reported bacteria detection limits from literature can be challenging due to factors other than the reporter system that influence detection capabilities. To advance the development of phage-based assays, a systematic comparison and understanding of the components are necessary. The objective of this study was to directly compare two common enzyme-mediated luminescence reporter systems, NanoLuc/Nano-Glo and alkaline phosphatase (ALP*)/DynaLight, for phage-based detection of bacteria. The detection limits of the purified enzymes were determined, as well as the expression levels and bacteria detection capabilities following engineering of the coding genes into T7 phage and infection of *E. coli* BL21. When comparing the sensitivity of the purified enzymes, NLuc/Nano-Glo enzyme/substrate system demonstrated a lower detection limit than ALP*/DynaLight. In addition, the expression of the NLuc reporter following phage infection of *E. coli* was greater than ALP*. The lower detection limit combined with the higher expression resulted in a greater than 100-fold increase in sensitivity for the NLuc/Nano-Glo^®^ reporter system compared to ALP*/DynaLight when used for the detection of *E. coli* in a model system. These findings provide a comparative analysis of two common reporter systems used for phage-based detection of bacteria and a foundational understanding of these systems for engineering future reporter phage assays.

## 1. Introduction

Bacteriophage (or phage) are a type of virus that infect and multiply within viable bacterial host cells [1]. Following infection, phage use their host to transcribe and translate the phage DNA, generating phage proteins and enzymes within a matter of hours or less [2,3,4]. These properties have been utilized to develop reporter phage assays to detect bacteria. Reporter phage-based assays are often built by genetically modifying phage genomes to express specific reporter proteins during infection. Detection of the expressed reporter proteins is used as a surrogate to indicate the presence of bacteria [5,6,7]. Reporters used in phages are typically enzymes that can give a detectable signal through a comprehensive range of output mechanisms such as electrochemical [8], colorimetry [5,7,9,10,11,12], fluorescence [13,14,15,16], or luminescence [5,17,18,19] upon interacting or reacting with an exogenous substrate. Of these methods, luminescence offers advantages over the other analytical techniques by having comparatively high sensitivity and extended linear responses [20].

Two common reporters employed in luminescence detection systems are alkaline phosphatase and NanoLuc luciferase. Alkaline Phosphatase (ALP) is a metalloid enzyme that hydrolyzes phosphoric esters [21]. The bacterial gene *PhoA* encodes the dimeric ALP for its phosphate metabolism [22,23,24]. ALP enzyme activity can be readily detected through several mechanisms such as electrochemical [25], colorimetric [10], fluorescence [14], and luminescence [7]. Recently, the efficacy of the enzyme has been improved by developing a double mutant ALP (ALP*), which increased the catalytic activity of the wild-type enzyme 40-fold [26]. Research shows that the gene of the mutant ALP* can be integrated into the genome of bacteriophage and expressed from *Escherichia coli* (*E. coli*) with conserved activity [7,10,14,27]. NanoLuc luciferase (NLuc) is a commercially available luciferase enzyme produced by Promega Corporation. The single subunit enzyme is obtained from the deep-sea shrimp *Oplophorus gracilirostris* and optimized to react with the synthetic substrate imidazopyrazinone (Nano-Glo^®^, Promega Corporation, Madison, WI, USA), resulting in a specific activity 150-fold greater than other class members such as firefly luciferase or *Renilla* luciferase [28]. The smaller size (19 kD), high thermostability, and high specific activity have made the NLuc enzyme a popular candidate as a reporter protein in gene recombination technology [19,29,30].

In addition to the specific enzyme/substrate reporter system, factors such as the species of phage, the reporter gene location, the presence/absence and type of signaling peptide, species, and strain of host cell, duration of infection, cell enrichment time, phage concentration, and the presence/absence and type of fusion tag can influence detection. As such, comparing reporter systems from literature results is challenging. In order to advance the development of phage-based assays, a systematic understanding and comparison of components are necessary. The objective of this study was to directly compare two enzyme-mediated luminescence reporter systems, NanoLuc/Nano-Glo and ALP*/DynaLight, for phage-based detection of bacteria in a model system. To achieve this objective, the detection limits of purified ALP* and Nluc, when used with their respective substrates, were determined. Genes coding for the enzymes were engineered into T7 phage, and expression levels and bacteria detection capabilities of the two enzymes were evaluated following infection of *E. coli* BL21.

## 2. Materials and Methods

### 2.1. ALP* and NLuc Constructs

**Scheme 1:**
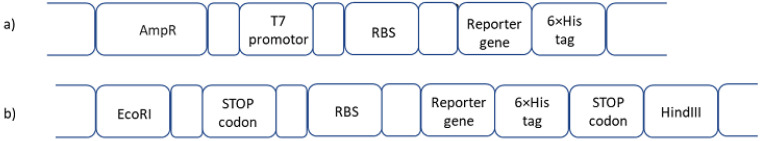
(**a**) Details of the reporter genes ALP* and NLuc constructs in the pET20b+ vector. The N-terminal pelB secretion signal was removed from the vector during plasmid synthesis. (**b**) Details of the reporter genes ALP* and NLuc constructs in the T7 phage genome.

NanoLuc Luciferase (NLuc) and double mutant (D135G/D330N) Alkaline Phosphatase (ALP*) enzymes were selected as the reporter enzymes to generate luminescence signals. Genbank accession numbers for NLuc and ALP* genes are JQ437370.1 and M29664.1, respectively. pET20b(+)NLuc plasmid was synthesized by GenScript Biotech. pET20b(+)ALP* was obtained from Professor Robert S. Haltiwanger, Complex Carbohydrate Research Center, University of Georgia. The pET20b(+) plasmids of ALP* and NLuc were designed without any secretory signal peptide sequences to retain the protein expression and activity at their native forms [31]. Therefore, the pelB sequence of the pET20b(+) vector was removed during plasmid synthesis. Both genes were designed to have C-terminal poly-histidine affinity tags (Scheme 1a).

### 2.2. Expression and Purification of ALP* and Nluc

The plasmid constructs from Genscript Biotech were transformed into NEB Express *E. coli* Bl21 cells. Transformed colonies were selected from LB agar plates with ampicillin at 100 μg/mL. A single colony was allowed to grow overnight, and 10 mL of the grown culture was added to 1 L of media until the OD reached 0.7. The 1 L culture of cells was induced with 200 μM IPTG and allowed to grow at 20 °C for another 18 h at 150 RPM. The cells were harvested by centrifugation at 5000 RPM for 45 min at 4 °C. The cell pellet was lysed using Bugbuster^®^ (MiliporeSigma, Burlington, MA, USA) solution diluted by 10% with equilibration buffer (50 mM sodium phosphate, 300 mM sodium chloride at pH 8) containing 10 mM imidazole, 1 mM PMSF, and 10 μg/mL DNAase. The cell suspension was mixed at 200 RPM for 30 min at room temperature, and the supernatant was obtained through centrifugation at 5000 RPM for 45 min at 4 °C. The supernatant was filtered using a 0.45 μm PVDF (Polyvinylidene Fluoride) filter and diluted proportionally with an equilibration buffer containing 10 mM imidazole. A glass chromatography column was packed with 500 μL of HisPur^™^ Ni-NTA resin (Thermo Fisher Scientific, Waltham, MA, USA) pre-equilibrated with equilibration buffer (50 mM sodium phosphate, 300 mM sodium chloride at pH 8) containing 10 mM imidazole. The diluted supernatant was passed through the packed column three times to allow maximum binding of the protein. The resin was washed with equilibration buffer containing 30 mM imidazole to remove non-specific protein. The reporter protein was eluted using an equilibration buffer containing 250 mM imidazole and concentrated using filter columns. ALP* protein was buffer exchanged with 50 mM Tris containing 10 mM magnesium chloride at pH 8, and NLuc protein was buffer exchanged with 50 mM phosphate buffer. The concentrated proteins were confirmed through SDS-PAGE, and the concentration was determined through the Bradford assay.

### 2.3. Enzyme Activity of Purified ALP* and NLuc

The purified NLuc and ALP* proteins were serially diluted to obtain solutions of 10–2 fmol/µL. To determine the enzyme activity of NLuc protein, Nano-Glo^®^ substrate was used, while for ALP* protein, DynaLight^™^ (Thermo Fisher Scientific, Waltham, MA, USA) substrate was used. For dilutions and blank samples, 50 mM phosphate buffer was used for NLuc protein, and 50 mM Tris containing 10 mM magnesium chloride was used for ALP* protein. A total of 30 µL of each protein dilution was mixed with 30 µL of the substrate solution. All reagents were allowed to come to room temperature, and the reactions were performed in black/brown microcentrifuge tubes. Twenty-five microliters of the reaction mixture were added to a 384-solid white microplate. The surrounding wells of each occupied well were kept empty to avoid well-to-well crosstalk signal. The Relative Luminescence Units of each sample were measured using Biotek^®^ (Synergy H1) microplate reader (BioTek, Vinooski, VT, USA) with luminescence mode, 135 gain setting, and a two-second integration time. Signal to noise (S/N) ratio was determined from the response at each concentration in relation to the response of the blank. Limit of detection (LoD) was estimated from regression analysis at concentrations corresponding to a S/N ratio of 3:1 using GraphPad^®^ Prism 8.2.0 (Graphpad Software, San Diego, CA, USA). Experiments were performed in triplicate.

### 2.4. Construction of Reporter Phages ALP* and Nluc

Commercially prepared T7Select^®^ cloning kit (MiliporeSigma, Burlington, MA, USA) was used as the phage cloning vector, which allowed the insertion of a reporter gene in substitution of a phage capsid gene [32]. The primer design and cloning of the reporters to phage vectors were carried out according to the guidelines in the T7Select^®^ cloning kit user manual. Genes coding for ALP* and NLuc were amplified using primers listed in Table 1 to prepare for the recombination with the phage genome (Scheme 1b) [33,34]. The restriction sites were used to obtain the specific sticky ends in the inserts for the ligation with the phage vector arms. The stop codons in the forward and reverse primers were employed to express the reporter individually and not as a fusion of the phage capsid protein [10]. The amplified PCR products were extracted using agarose gel electrophoresis, where bands were observed for ALP* insert (~1350 bp) and NLuc insert (~531 bp) relative to the 1 kb DNA ladder and confirmed using Sanger sequencing. The extracted and purified PCR products of the reporter gene inserts (0.04 pmol/µL) were digested with EcoRI and HindIII restriction enzymes. The ligation reaction was carried out using the digested reporter genes (1.5 µL) and the T7Select^®^ phage vector arms (1 µL of 0.02 pmol/µL) to have the reporter gene to vector arms at a 3:1 molar ratio. The finished ligation reaction was directly added to the T7Select^®^ Packaging Extracts provided by the cloning kit for in vitro packaging. The packaged phages were confirmed for viability and amplified through the double agar overlay plaque assay. The DNA of several plaques was extracted, amplified by PCR, and subjected to Sanger sequencing to confirm the presence of the reporter gene insert. The background reporter protein of the phage stock was removed by passing the stock through a nickel-NTA resin-packed column until a minimal luminescence signal was detected. Concentrations of the final phage stocks were determined using a double agar overlay plaque assay.

### 2.5. Quantitation of Reporter Protein through the Phage Assay

A total of 45 mL of the diluted phage solution (10^7^ PFU/mL) was mixed with 4.5 mL of a diluted overnight culture of *E. coli* BL21 cells (10^7^ CFU/mL) in a 50 mL centrifuge tube. A total of 45 mL of the same phage solution was mixed with 4.5 mL of LB as the control. The tubes were incubated at 37 °C at 175 RPM for 2 h. After the incubation, the phage-*E. coli* mixtures were passed through a glass chromatography column packed with 500 μL of HisPur^™^ Ni-NTA resin. The resin was pre-equilibrated with equilibration buffer (50 mM sodium phosphate, 300 mM sodium chloride at pH 8) containing 10 mM imidazole. The column was washed with equilibration buffer containing 30 mM imidazole. The reporter protein was eluted using an equilibration buffer containing 250 mM imidazole and concentrated using filter units. ALP* protein was buffer exchanged with 50 mM Tris containing 10 mM magnesium chloride at pH 8, and NLuc protein was buffer exchanged with 50 mM phosphate buffer. NanoOrange^™^ Protein Quantitation Kit (Thermo Fisher Scientific, Waltham, MA, USA) protocol was followed to quantify the amount of reporter enzyme of each engineered phage. Briefly, 100 μL of each concentrated protein was added to 2.4 mL of NanoOrange^™^ working stock dye, heated for denaturation of the protein, and cooled before measuring the fluorescence. The relative fluorescence units (RFU) were measured using a BioTek^®^ plate reader with an excitation at 485 nm and emission capturing at 590 nm. A standard curve was generated using bovine serum albumin protein (BSA; 0.1 ng/mL–2 mg/mL) with NanoOrange^™^ dye to determine the concentrations of the reporter protein. The protein amounts obtained from the control were subtracted from the experiment to get the total amount of reporter proteins expressed through the engineered phages upon infection. Molecular weights of 19 kD for NLuc reporter and 96 kD for ALP* reporter were taken when calculating molecules of protein expressed per *E. coli* cell. All experiments were performed in triplicate, and the data were expressed as the mean ± standard deviation. The statistical analysis through GraphPad^®^ Prism software was used to perform statistics with a *p* < 0.05 indicating a statistically significant difference.

### 2.6. Limit of Detection of Reporter Phage for E. coli

Reporter phage stocks NLuc and ALP* were diluted to obtain 10^9^ PFU/mL. An overnight culture of *E. coli* BL21 cells was serially diluted to obtain 10 to 10^10^ CFU/mL cultures. A total of 800 μL of the 10^9^ PFU/mL phage stock was mixed with 80 μL of each *E. coli* BL21 dilution. A total of 800 μL of the 10^9^ PFU/mL phage stock was mixed with 80 μL of LB as the blank experiment. The mixtures were incubated at 37 °C with 175 RPM for 2 h to allow complete infection of *E. coli* cells and expression of the reporter protein. Following incubation, the samples were centrifuged (3000× *g*, 3 min) to pellet any cell debris. The supernatant of each dilution was collected to determine the luminescence activity of each reporter according to the procedure in Section 2.3 [35].

## 3. Results

### 3.1. Expression and Purification of ALP* and NLuc

**Figure 1:**
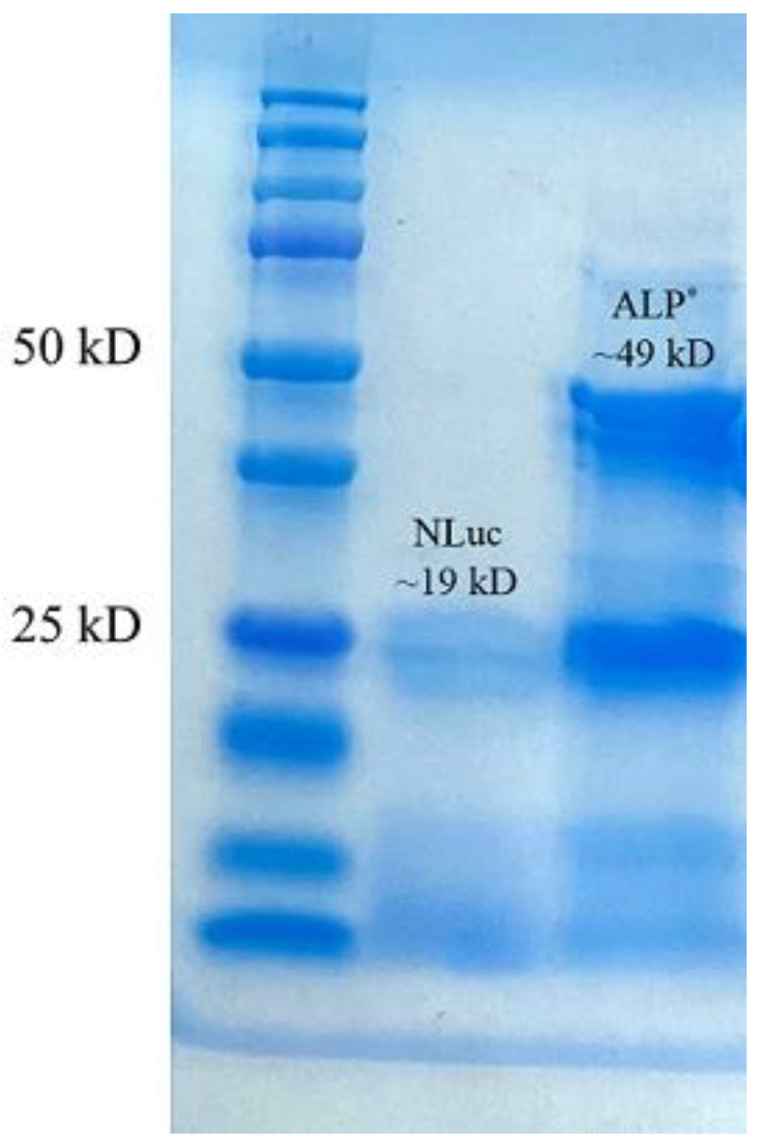
Detection of NLuc and ALP* in the soluble fraction of *E. coli* Bl21.

To determine the detection limits of ALP* and NLuc with their respective substrates in solution, the two enzymes were cloned, expressed, and purified. Both enzymes could be expressed in the soluble fraction of the cell lysate (Figure 1). The monomeric NLuc protein showed a band along its respective size (19 kD), and the dimeric ALP* showed a band at its single subunit size of ~48 kD in the SDS-PAGE gel. Though ALP* has an active site in each subunit, the protein’s high catalytic activity depends on the formation of a dimer [36]. The SDS-PAGE gel confirmed previous reports that NLuc and ALP* proteins are expressed in the bacterial cytoplasm [37,38]. While genes for both reporters were designed without sequences to promote secretion in the periplasm, ALP* showed protein expression in the cell medium in the absence of secretory signals. This unexpected phenomenon could be attributed to undefined signal peptides within the gene sequence [39]. Following Ni-NTA column purification, 0.25 mg of ALP* and 0.3 mg of NLuc could be recovered from 1 L cultures.

### 3.2. Enzyme Activity of Purified ALP* and NLuc

**Figure 2:**
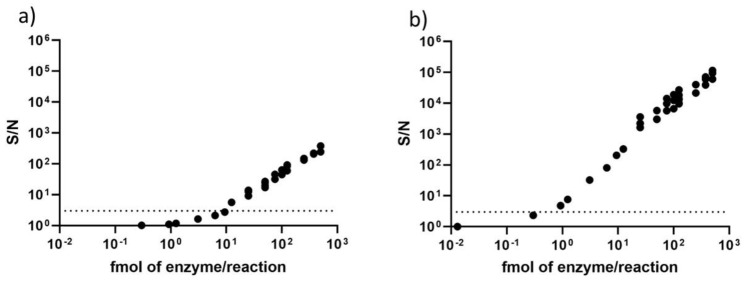
Signal-to-noise ratio (S/N) versus the amount of enzyme. (**a**) ALP*/DynaLight and (**b**) NLuc/Nano-Glo. S/N of 3:1 is indicated by a dashed line (n = 3).

A linear response was observed as a function of protein concentration for both purified enzymes in combination with their luminescent substrates. Based on an S/N ratio of 3:1 and molecular weights 96 kD and 19 kD assumed for ALP* and NLuc, the detection limits for the defined assays were estimated at 0.31–0.42 fmol/μL (9.4–12.5 fmol/reaction) for ALP*/DynaLight^™^ and 0.01–0.03 fmol/μL (0.3–0.93 fmol/reaction) NLuc/Nano-Glo^®^ (Figure 2).

### 3.3. Construction of Reporter Phages ALP* and NLuc

**Figure A1:**
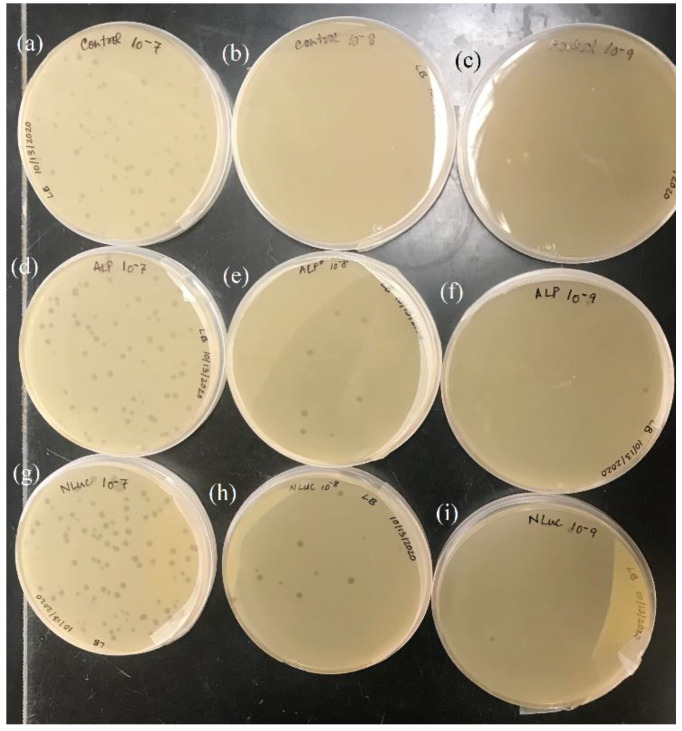
Plaques produced by Control phage (**a**–**c**), ALP* phage (**d**–**f**), and NLuc phage (**g**–**i**). Each row is dedicated to a recombinant phage and the three dilutions used for each phage (10^−7^ PFU/mL to 10^−9^ PFU/mL) show from left to right.

Recombinant T7 phages with reporter gene inserts were amplified using an exponentially growing *E. coli* BL21 cells culture to ensure efficient phage infection and replication. Visible plaques were observed in the plaque assays, confirming the reporter genes’ successful ligation into the phage genome. The lysis time of the cell culture and plaque sizes were similar to the control phage synthesized by the T7Select^®^ cloning kit (Figure A1), suggesting that the gene inserts did not affect the fitness of the recombinant phages. PCR amplification of the extracted DNA from a single plaque and agarose gel electrophoresis confirmed the presence of the reporter genes in each engineered phage. Sanger sequencing further confirmed no significant mutations in the reporter genes. Phage stock concentrations were calculated to be 5.4 × 10^10^ PFU/mL and 5.5 × 10^10^ PFU/mL for T7_ALP*_ and T7_NLuc_, respectively.

### 3.4. Quantitation of Reporter Protein through the Phage Assay

**Figure 3:**
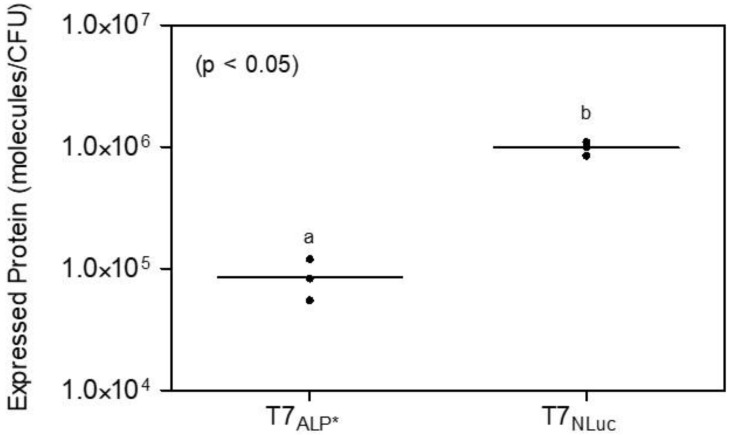
Reporter protein expressed following phage infection of *E. coli* BL21 cells. Different letters indicate statistically significant differences (*p* < 0.05) in protein expression (n = 3).

While the enzyme’s catalytic activity, in combination with the sensitivity of the specific substrate, is essential to the detection capabilities, protein expression levels through the host must also be considered when selecting a reporter for phage assays [8,16,31]. To determine expression, infection of the bacterial cells was maintained using a 10-fold concentration of phage relative to CFUs of *E. coli*. With the assumptions that the infection of all *E. coli* cells by at least one recombinant phage and that the purified protein was entirely reporter, the average molecules of reporter expressed per colony forming unit (CFU) was calculated to be ~9.8 × 10^5^ molecules (~1.6 attomoles) for T7_NLuc_ and ~8.6 × 10^4^ molecules (0.14 attomoles) for T7_ALP*_ * (Figure 3). These results indicate that T7_NLuc_ is able to express significantly more (*p* = 0.0132) reporter molecules/CFU than T7_ALP*_.

### 3.5. Limit of Detection of Reporter Phage for E. coli

**Figure 4:**
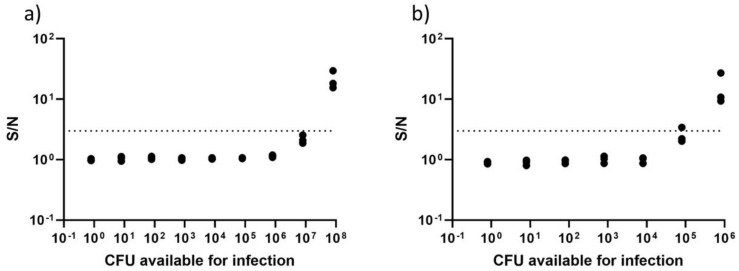
Signal to noise ratio versus colony forming units (CFU) available for infection in 880 mL. (**a**) T7_ALP*_/DynaLight and (**b**) T7_NLuc_/Nano-Glo. S/N of 3:1 is indicated by a dashed line. (n = 3).

A series of overnight grown *E. coli* BL21 concentrations were prepared to determine the limit of detection when using the recombinant phages in a model system. Overnight bacterial cultures were used to mimic the stationary phase *E. coli* that would be present in field samples. A fixed and higher phage concentration was used to achieve infection of all *E. coli* cells and maintain the initial culture concentrations. Based on an S/N ratio of 3:1, the limit of detection of T7_ALP*_ was determined to be within 8 × 10^7^ CFU/mL–8 × 10^8^ CFU/mL, while the limit of detection for T7_NLuc_ was within 8 × 10^5^ CFU/mL–8 × 10^6^ CFU/mL relative to the phage-only negative control (Figure 4).

## 4. Discussion

The genome of T7 phage can be genetically engineered to successfully express catalytically-active alkaline phosphatase and NanoLuc reporter enzymes following infection of *E. coli* BL21 cells. While both reporter phages can be used for the luminescence-based detection of *E. coli* in a model system, T7_NLuc_ is superior to T7_ALP*_ with respect to bacteria detection sensitivity. The lower limit of detection attributed to T7_NLuc_ when using Nano-Glo^®^ is attributed to a more sensitive enzyme/substrate system and a higher protein expression level. With respect to the enzyme/substrate system, NLuc/Nano-Glo^®^ demonstrated a lower background, a steeper slope, and a detection threshold ~10-fold greater than the ALP*/DynaLight™ system, suggesting the NLuc/Nano-Glo^®^ is the more sensitive luminescent system [40,41]. Increased protein expression is likely a function of the smaller size of NLuc phage (~531 bp/~19 kD) compared to ALP* (~1350 bp/~96 kD). A smaller sequence may enhance transcription, translation, and subsequent mean abundance of protein per cell [42]. These expression values are aligned with the reported expression of a maltose binding protein-tobacco etch virus (TEV) protease fusion protein (~2048 bp/~72 kD) that was estimated to be ~2 × 10^5^ molecules per host cell [8,16]. However, as evident by the SDS-PAGE gel, His-tag purification of ALP* resulted in additional proteins collected in the purified ALP*. We hypothesized that these additional bands to be proteolyzed ALP or native *E. coli* proteins that had an affinity to Ni-NTA. The additional proteins in the purified ALP* could risk overestimating the total expressed reporter of ALP* phage during the assay procedure. A more specific His-tag purification system of the reporter such as using a cobalt-based resin, may improve the purity of the expressed ALP* and provide more accurate quantitation of the phage expressed reporter.

While the results indicate that direct detection of *E. coli* is possible using T7 phage with an NLuc or ALP* reporter, modifying the reporter or assay system may enhance sensitivity. For example, Pulkkinen and others engineered a T7 phage that would express an NLuc reporter upon *E. coli* infection [19]. The NLuc reporter was modified to include a PelB-leader sequence to foster expression and a carbohydrate-binding module (CBM) to enable downstream immobilization. Using this system, a detection limit of 47 CFU per well was achieved. Hinkley and others also utilize an NLuc-CBM fusion reporter and T7 phage to achieve a limit of detection of less than 10 CFU/mL [43]. NLuc reporter was fused with CBM to concentrate the expressed NLuc reporter on microcrystalline cellulose. In addition, the detection system included an initial growth step of 1–5 h to increase the number of bacterial cells in the sample. ALP* has also been fused with CBM to enable concentration of the reporter following host infection. Singh and others were able to achieve detection limits of <10 CFU/mL using these concentration strategies along with enrichment steps [4]. These examples demonstrate the potential of reporter modifications to improve phage-based bacterial detection systems. However, other assay factors, such as the incubation time, incubation volume, reaction volume, phage type, phage concentration, and readout method, can also influence the sensitivity (Table 2).

Collectively, the attributes of T7_Nluc_ enable an approximately 100-fold enhancement in sensitivity for the detection of *E. coli* BL21 when compared to T7_ALP*_. These findings provide a comparative analysis of two common reporter systems used for phage-based detection of bacteria and a foundational understanding of the components for systematic engineering of future phage-based detection assays.

## Figures and Tables

**Table 1:** Primer sets used to generate and NLuc and ALP* gene inserts for the ligation with the T7Select^®^ phage vector arms.

NLuc	Restriction Site	RBS	STOP	START	Primer
Forward Primer	EcoRI GAATTC	AGGAGG	TGATGA	ATG	5′CGGGAATTCATGATGAGCGAGGAGGGCGATGATGGTCTTCACACTC 3′
Reverse Primer	HindIII AAGCTT		TGA		5′CGGAAGCTTCTAGTGGTGGTGGTGGTGGTGCGCCAG 3′
ALP*	**Restriction Site**	**RBS**	**STOP**	**START**	**Primer**
Forward Primer	EcoRI GAATTC	AGGAGG	TGATGA	ATG	5′CGGCGGAATTCATGATGAGCGAGGAGGCGCATGCGTACACCGGAAATGCCG 3′
Reverse Primer	HindIII AAGCTT		TGA		5′CGGAAGCTTTCAGTGGTGGTGGTGGTGGTGCTCGAGTTTCAG 3′

**Table 2:** Summary of Selected NLuc and ALP* Reporter phage-based detection assays.

Ref.	Type of Phage	*E. coli* Strain	Reporter Modification	Phage Available for Infection	Pre/Enrich-ment of *E. coli*	Incubation Time-Volume	Reporter Concentration	Reaction Volume	Detection Matrix	Detection Method	Definition of the Detection Limit- Total Assay Time
[4]	T7_ALP*	BL21 derivative	CBM(Cex)	1:10 CFU:PFU	4 h	2 h- 100 mL	Magnetic cellulose particles	60 µL	LB	Abs	^3^LOQ: <10 CFU/100 mL-8 h
[7]	T7_ALP*	BL21	His-tag	10^7^ PFU/mL	-	16 h-	-	50 µL	LB	Abs	^7^LOD: ∼1 × 10^5^ CFU/mL
[10]	T7_NLuc	BL21	CBM2a/pelB	2 × 10^9^ PFU	8–12 h	1.5 h- 2 mL	Cellulose filter	~300 µL	Drinking water	Abs/LU	Matched EPA results
[19]	T7_NLuc	BL21	CBM/pelB	10^6^ PFU	1 h	2 h- 1 mL	-	200 µL	LB	LU	^6^Detectable signal: 5 × 10^2^ CFU/mL-2 h
[43]	T7_NLuc	BL21	CBM/pelB	10^9^ PFU	1 h (pre-)	1.5 h-126 mL	Micro-crystalline cellulose	100 µL	Drinking water	LU	^4^LOD: <10 CFU/mL-3 h
[44]	T7_NLuc	BL21	CBM	1.25 × 10^8^ PFU	-	3 h- 0.25 mL	Nitro-cellulose membrane	75 µL	Drinking water	LU	^1^LOD: 4.1 CFU-5.5 h
[45]	T7_NLuc	ECOR13	CBM	10^7^ PFU/mL	3 h (pre-)	1.5 h-	Cellulose filter	-	Drinking water	LU	^2^LOD: <20 CFU-5 h
[46]	T4_NLuc	ECOR13	CBM	10^7-6^ PFU	3 h (pre-)	3 h- 0.5 mL	Cellulose filter	50 µL	Drinking water	LU	^5^LOD: <10 CFU/100 mL-7 h
[47]	K1E_NLuc	K1	-	10^6^ PFU	-	1 h- 0.1 mL	-	-	LB	LU	^8^LLOD: 8 CFU/well-1 h

Definition of Limit of Detection used by each assay. ^1^LOD—Negative control (phage only) plus three standard deviations. ^2^LOD—Mean blank plus three times the standard deviation of the blank. ^3^LOQ: Limit of Quantitation. Ten times the standard deviation of the mean blank divided by the regression slope. ^4^LOD—Sum of the negative control and three standard deviations. ^5^LOD—Three standard deviations above mean baseline luminescence. ^6^Detectable Signal—Negative control (phage only) and its standard deviation was considered. ^7^LOD— Positive signals above background. ^8^LLOD: Lower Limit of Detection. Addition of three times the standard deviation of the average measurement of the negative control to the average negative control value.

## Data Availability

The data presented in this study are available on request from the corresponding author.
